# MACF1 alleviates aging‐related osteoporosis via HES1

**DOI:** 10.1111/jcmm.16579

**Published:** 2021-06-15

**Authors:** Chong Yin, Ye Tian, Lifang Hu, Yang Yu, Zixiang Wu, Yan Zhang, Xue Wang, Zhiping Miao, Airong Qian

**Affiliations:** ^1^ Lab for Bone Metabolism Xi'an Key Laboratory of Special Medicine and Health Engineering Key Lab for Space Biosciences and Biotechnology Research Center for Special Medicine and Health Systems Engineering NPU‐UAB Joint Laboratory for Bone Metabolism School of Life Sciences Northwestern Polytechnical University Xi'an China; ^2^ Lab of Epigenetics and RNA Therapy Department of Clinical Laboratory, Academician (Expert) Workstation Affiliated Hospital of North Sichuan Medical College Nanchong China; ^3^ Department of Laboratory Medicine North Sichuan Medical College Nanchong China; ^4^ Translational Medicine Research Center North Sichuan Medical College Nanchong China; ^5^ Tianjin Key Laboratory on Technologies Enabling Development Clinical Therapeutics and Diagnostics (Theranostics) School of Pharmacy Tianjin Medical University Tianjin China

**Keywords:** ageing, bone formation, HES1, MACF1, osteoporosis

## Abstract

Ageing‐related osteoporosis is becoming an emerging threat to human health along with the ageing of human population. The decreased rate of osteogenic differentiation and bone formation is the major cause of ageing‐related osteoporosis. Microtubule actin cross‐linking factor 1 (MACF1) is an important cytoskeletal factor that promotes osteogenic differentiation and bone formation. However, the relationship between MACF1 expression and ageing‐related osteoporosis remains unclear. This study has investigated the expression pattern of MACF1 in bone tissues of ageing‐related osteoporosis patients and ageing mice. The study has further elucidated the mechanism of MACF1 promoting bone formation by inhibiting HES1 expression and activity. Moreover, the therapeutic effect of MACF1 on ageing‐related osteoporosis and post‐menopausal osteoporosis was evaluated through in situ injection of the MACF1 overexpression plasmid. The study supplemented the molecular mechanisms between ageing and bone formation, and provided novel targets and potential therapeutic strategy for ageing‐related osteoporosis.

## 1 INTRODUCTION

Ageing‐related osteoporosis is a high incidence disease, which was identified as deterioration in bone mass and bone strength, and it would increase the risk of fracture.^1^ The incidence of ageing‐related osteoporosis is enhancing along with the ageing of human population and had become a major threat to the health of senile human. The causes of ageing‐related osteoporosis are variable and complex, and researchers have found that osteogenic differentiation is inhibited via multiple signalling pathways during ageing processes both in male population and in post‐menopausal female population, which resulted in declined bone formation and osteoporosis gradually.^2^, ^3^ Among all the ageing‐related signalling pathways, Wnt/β‐catenin pathway and Notch signalling pathway have been reported to highly correlate with ageing‐related osteoporosis.^4^, ^5^, ^6^

Wnt/β‐catenin pathway is one of the most essential pathways regulating osteogenesis, it activates the expression of multiple osteogenic factors by its transcription factor TCF7/LEF1.^3^, ^4^ Notch signalling pathway also played an important role on osteoblast differentiation. Hairy/enhancer of split 1 (HES1) is the transcription factor, which functions as the downstream of the Notch signalling pathway and inhibits bone formation,^6^ and Hilton's study proved that HES1 physically interacted with osteogenic factor RUNX2 and diminished its activity, thus inhibited osteoblast differentiation.^7^

Microtubule actin cross‐linking factor 1 (MACF1) also named ACF7 (actin cross‐linking factor 7), is a member of the plakin family.^8^ It is a 600 kDa cytoskeletal protein that cross‐links with actin and microtubules, and MACF1 regulates multiple physiological and pathological processes.^8^, ^9^, ^10^, ^11^, ^12^, ^13^ In cellular level, MACF1 regulates intracellular material transportation and thus plays an important role in signalling pathways. Chen has found MACF1 participates in Wnt/β‐catenin pathway by facilitating β‐catenin to dissociate from the β‐catenin/AXIN/APC complex and thus transport to cell nucleus.^14^ Burgo's study has found that MACF1 manipulates vesicles transporting from the trans‐Golgi network to cell periphery.^15^ Previous studies have proved that MACF1 promotes osteogenic differentiation and bone formation.^16^, ^17^, ^18^, ^19^ In Hu's study, MACF1 promoted osteoblast differentiation by stabilizing β‐catenin.^16^ In Zhang's study, MACF1 overexpression plasmid was transfected to osteoblast in vitro and in vivo, thus enhanced osteoblast differentiation and bone formation.^17^ These studies implied that MACF1 plays an essential role on regulating osteogenesis; however, reports on the effects of MACF1 on ageing‐related osteoporosis are still limited.

In this study, we explored the expression levels of MACF1 in femur tissues of ageing patients and ageing mice. And we have found that MACF1 expression was associated with the reduction in osteogenesis in ageing‐related osteoporosis. The mechanism of MACF1 promoting bone formation was also investigated. The results implied that HES1 expression and activity might involve in aging related osteoporosis. Enhancing the expression of MACF1 by plasmids would efficiently rescue bone formation in ageing‐related osteoporosis and post‐menopausal osteoporosis mice.

## 2 MATERIALS AND METHODS

### 2.1 Cell culture, human sample and mice model

Murine preosteoblast MC3T3‐E1 cell line was generously provided by Dr Hong Zhou (The University of Sydney, Sydney, Australia). MC3T3‐E1 cell line and mice primary bone marrow mesenchymal stem cells (BMSCs) were cultured in culture medium containing alpha‐modified Eagle's medium (α‐MEM; Gibco, 11900‐024) supplemented with 10% foetal bovine serum (FBS; Biological Industries, 04‐001‐1A, Kibbutz Beit Haemek, Israel), 1% L‐glutamine (Sigma, G8540, St Louis, MO), 1% penicillin (Amresco, 0242, Solon, OH) and streptomycin (Amresco, 0382, Solon, OH). Cell cultures were maintained at a humidified, 37°C, 5% CO_2_ incubator (Thermo Fisher Scientific, Waltham, MA). For osteogenic differentiation treatment, cells at density of 100% were induced by osteogenic medium with α‐MEM, 10% FBS, 1Mm dexamethasone (Sigma, D4902), 1% β‐glycerophosphate (Sigma, G9422), 1% ascorbic acid (Sigma, A7631) and 1% L‐glutamine. The osteogenic medium was replaced every 2 days.

18 human femur samples were collected from Xi'an Honghui Hospital. Inclusion criteria for the study were healthy post‐menopausal women between 60 and 90 years of age with fracture caused by falling without obvious violence, and undergoing hip arthroplasty operation within one week after fracture. Exclusion criteria included liver and kidney diseases, osteoarthritis, systemic inflammatory diseases, endocrine diseases, malignancy, diabetes, hormone therapy in recent 3 months or other severe diseases in the previous 5 years. We collected trabecular bone tissue at the distal end of the fracture site of femoral head during hip replacement operation, tissue samples were processed for RT‐PCR, Western blot and IHC, samples for RT‐PCR were stored in RNAlater solution (Qiagen, 76 106, Hilden, Germany), and all samples were stored at −80°C until analysis. This study was approved by Biomedical Research Ethics Committee of Hong Hui Hospital (3 March 2017) and Institution Review Board of the Northwestern Polytechnical University (12 September 2016). All participants were provided with informed written consent before their participation in this study.

Ageing and ovariectomized (OVX) mice were adopted to construct the osteoporosis model. All mice were purchased from the Laboratory Animal Center of the Fourth Military Medical University (Xi'an, China). For ageing male mice model, eighty 6‐month‐old C57BL/6 mice were maintained under standard animal housing conditions (12‐h light, 12‐h dark cycles and free access to food and water). 61 mice were kept until 21 months old and selected as ageing group, whereas 19 mice were used as normal group. 38 mice (19 from ageing male group and 19 from normal group) were killed, and femurs were collected and processed for bone marrow mesenchymal stem cell isolation (n = 5/group), RT‐PCR (n = 5/group), Western blot (n = 3/group), immunohistochemical staining (n = 3/group) and RNA‐seq (n = 3/group). The other 42 mice were processed for MACF1 overexpression plasmid (PEGFP‐C1A‐ACF7) in vivo transfection. For ageing female mice model, six 6‐month‐old C57BL/6 mice were maintained under standard animal housing conditions. Three mice were kept until 21 months old and used as ageing group, and other 3 mice were used as normal group. All mice were killed, and femurs were collected for RNA‐seq (n = 3/group).

For OVX mouse model, forty‐two 2‐month‐old female C57BL/6 mice were maintained under standard animal housing conditions. The mice were ovariectomized or sham‐operated at 3 months of age. Mice were used to investigate MACF1 overexpression plasmid therapeutic effect and were killed 35 days after surgery (4 months of age), and calvarials were collected. Euthanasia was performed by CO_2_. All animal experiments were performed in accordance with the recommendation of ‘the Guiding Principles for the Care and Use of Laboratory Animals’ (the Institutional Experimental Animal Committee of Northwestern Polytechnical University, Xi'an, China), and all experimental procedures were approved by the Institutional Experimental Animal Committee of Northwestern Polytechnical University, Xi'an, China. For all procedures involving animals, all efforts were made to reduce the number of the mice used and their suffering.

### 2.2 Isolation of bone marrow mesenchymal stem cells (BMSCs)

Mice bone marrow mesenchymal stem cells were isolated for investigating the effect of ageing on osteogenic differentiation. 6‐ and 21‐month‐old mouse were killed, and mice femurs were immediately harvested. Attached soft tissues on femoral bones were carefully removed. Bone marrow was washed and collected by flushing several times with phosphate‐buffered saline (PBS) using a 25G syringe needle. The collected PBS with bone marrow was centrifuged (1200 g, 8 minutes) and dissociated by culture medium (α‐MEM, Gibco supplemented with 10% foetal bovine serum, 1% L‐glutamine, 1% penicillin and streptomycin) using a 29G syringe needle. The suspension was cultured in a 60‐mm plate for 3 hours (37°C, 5% CO_2_) followed by careful wash with culture medium. Cells were cultured for another 36 hours with culture medium changed every 12 hours. The cells were transferred into a new plate as the 1st passage cells. Third passage cells were used for characterization and experiments.

### 2.3 RNA‐seq analysis

RNA‐seq was used to assess expression levels of ageing‐related mRNAs. Total RNA was extracted from 6 to 21 months of both male and female mouse femurs, respectively, using TRIzol Reagent (Invitrogen, 15 596 018). After extracting RNA from cells, the quality and quantity of the purified RNA were determined by measuring the absorbance at 260 nm/280 nm (A260/A280) using NanoDrop OneC (Thermo Fisher Scientific). RNA integrity was further verified by 1.5% agarose gel electrophoresis. RNA‐seq was performed at Annoroad Gene Technology Co. Ltd. (Beijing). The fold change in each deferentially expressed RNA was obtained by log_2_ (normalized intensity of treat/normalized intensity of control).^20^

### 2.4 Real‐time PCR

RT‐PCR was used to assess expression levels of MACF1 and osteogenic markers. Total RNA was extracted from femur tissues or cultural cells using TRIzol reagent. Femur tissues were harvested, washed by RNAlater solution, grind with liquid nitrogen and then digested by TRIzol reagent. 1μg of total RNA was used for cDNA synthesis using One Step PrimeScript RT Reagent Kit (Takara, RR037A, Dalian, China). Quantitative PCR amplification was performed with the Thermal Cycler C‐1000 Touch System (Bio‐Rad CFX Manager, Hercules, CA) and SYBR Premix Ex Taq II Kit (Takara, RR820A). *Gapdh* was used as internal control gene. The quantitative PCR conditions included initial denaturation step at 95°C for 30 seconds, followed by 42 cycles at 95°C for 10s, 60°C for 30s and 72°C for 5s. Data were calculated using the comparative Ct method (2^−ΔΔCt^) and expressed as fold change compared with corresponding control. Primers (sequences; Table 1) were synthesized by Sangon Int (Shanghai, China).

**TABLE 1:** Primer sequences for qRT‐PCR

Target gene	Sequences (5’→3’)
Human *MACF1*‐Forward	AACCAAGGCAACCCATTCTT
Human *MACF1*‐Reverse	ACTCTGCTCGCTCCAGTTTC
Human *Ocn*‐Forward	GGTGCAGCCTTTGTGTCCAAGC
Human *Ocn*‐Reverse	GTCAGCCAACTCGTCACAGTCC
Human *Osterix*‐Forward	CGGCAAGAGGTTCACTCGTTCG
Human *Osterix*‐Reverse	TGGAGCAGAGCAGGCAGGTG
Human *Hes1*‐Forward	ACGTGCGAGGGCGTTAATAC
Human *Hes1*‐Reverse	GGGGTAGGTCATGGCATTGA
Human *Gapdh*‐Forward	CATGGAGAAGGCTGGGGCTC
Human *Gapdh*‐Reverse	CACTGACACGTTGGCAGTGG
Mouse *MACF1*‐Forward	GAAAACATTCACCAAGTGGGTCAAC
Mouse *MACF1*‐Reverse	TGTCCATCCCGAAGGTCTTCATAG
Mouse *Ocn*‐Forward	GAAGGCAACAGTCGATTCACC
Mouse *Ocn*‐Reverse	GACTGTCTTGCCCCAAGTTCC
Mouse *Osterix*‐Forward	CCCTTCCCTCACTCATTTCC
Mouse *Osterix*‐Reverse	CAACCGCCTTGGGCTTAT
Mouse *Hes1*‐Forward	AAGAAAGATAGCTCGCGGCA
Mouse *Hes1*‐Reverse	CCTCGGTATTAACGCCCTCG
Mouse *P16*‐Forward	GCTTCTCACCTCGCTTGTCACAG
Mouse *P16*‐Reverse	CGGGATCGCACGAACTTCACC
Mouse *Alp*‐Forward	GTTGCCAAGCTGGGAAGAACAC
Mouse *Alp*‐Reverse	CCCACCCCGCTATTCCAAAC
Mouse *Runx2*‐Forward	CGCCCCTCCCTGAACTCT
Mouse *Runx2*‐Reverse	TGCCTGCCTGGGATCTGTA
Mouse *Col I*‐Forward	GAAGGCAACAGTCGATTCACC
Mouse *Col I*‐Reverse	GACTGTCTTGCCCCAAGTTCC
Mouse *Gapdh*‐Forward	TGCACCACCAACTGCTTAG
Mouse *Gapdh*‐Reverse	GGATGCAGGGATGATGTTC

### 2.5 Western blot

For the detection of protein levels, Western blot analysis was performed as previously described.^21^ Protein samples from femur tissue or cultural cells were extracted. For femur tissue from mouse and patients, bone marrow and attached soft tissue were removed carefully removed. The tissue was washed by 1% protease inhibitor cocktail (Calbiochem, 539 134, Darmstadt, Germany) and then grind by mortar with liquid nitrogen and digested by cell lysis buffer (Beyotime, P0013, Haimen, China) with 1% protease inhibitor cocktail on ice for 30 mins. As for cultural cells, the cells were washed three times by cold PBS and then digested by cell lysis buffer with 1% protease inhibitor cocktail. Protein concentrations were analysed by BCA Protein Assay Kit (Thermo Fisher Scientific, 23 225). 100 ng of proteins for each sample was subjected to SDS‐PAGE using 6% stacking gel and 10% separating gel, 140V, 30 mins, and transferred (400 mA, 2 hours) to nitrocellulose filter membranes (Pall, 66 485, Port Washington, NY). Membranes were blocked with 5% skimmed milk (BD Biosciences, 232 100, Franklin Lakes, NJ) for 1 hour at room temperature and then incubated with primary antibodies at 4°C overnight with the following primary antibodies: MACF1 (Rabbit pAb, 1:500; Abcam, ab117418, Cambridge, UK), OCN (Rabbit pAb, 1:500; Proteintech, 23418‐1‐AP, Hubei, China), OSTERIX (Rabbit pAb, 1:500; Proteintech, 12593‐1‐AP), HES1 (Rabbit pAb, 1:500; Bioss, bs‐2972R‐1, Beijing, China) and GAPDH (Rabbit pAb, 1:1000; Proteintech, 10494‐1‐AP). Blots were then incubated with HRP‐labelled secondary antibody (1:2000; CWBIO, CW0103; Beijing, China) and visualized using chemiluminescence detection system (Thermo Fisher Scientific, NCI5080). Protein bands were exposed to X‐ray film (Kodak, 6535876). GAPDH was adopted as internal control.

### 2.6 Immunohistochemical staining

To investigate the effect of ageing on osteogenic gene and transcription factor expression levels, and to determine the rescue effect of MACF1 on osteoporosis, immunohistochemical staining analysis was performed as previously described.^22^ Human and mouse femur samples, along with mouse calvarias, were dissected and fixed in 4% paraformaldehyde, and decalcified in 17% ethylene diamine tetraacetic acid (EDTA; Sigma, E9884) for 50, 30 and 15 days, respectively. Decalcified bone samples were dehydrated using graded ethanol, vitrified by dimethylbenzene and embedded in paraffin (Huayong, Shanghai, China). Sections (5 μm in thickness) were dewaxed, immersed in the distilled water, blocked in 5% goat serum (CWBIO, CW0130) in PBS and then incubated overnight at 4°C with primary antibodies against MACF1 (Rabbit pAb, 1:100; Abcam, ab117418), OCN (Rabbit pAb, 1:100; Proteintech, 23418‐1‐AP), OSTERIX (Rabbit pAb, 1:100; Proteintech, 12593‐1‐AP) and HES1 (Rabbit pAb, 1:100; Bioss, bs‐2972R‐1), respectively. Following three washes in PBS, the sections were labelled with HRP‐labelled secondary antibody for 1.5h at room temperature and developed for colour reaction using diaminobenzidine (DAB, CWBIO, CW2068) and haematoxylin counterstain. Slides were scanned by Aperio AT2 Digital Pathology Scanner (Leica), and protein immunostaining intensities on top surface of the calvarias were analysed by Image‐Pro Plus 6.0 software.

### 2.7 Overexpression and knockdown of MACF1

MACF1 overexpression cells were constructed by transfection of plasmid PEGFP‐C1A‐ACF7 as previously described,^21^ with PEGFP‐C1–transfected cells as control. MC3T3‐E1 cells (1 × 10^7^ per well) or primary BMSCs (5 × 10^6^ per well) were electroplated (1800V, 30ms) with the MACF1 overexpression plasmid PEGFP‐C1A‐ACF7 or normal plasmid PEGFP‐C1A by using Neon Transfection System (Invitrogen) according to manufacturer's instructions. After the electroporation, cells were seeded into a 6‐well plate with 10ml α‐MEM and cultured for 6h. Adherent cells were then washed by 2ml α‐MEM two times, and medium was changed to antibiotic‐free culture medium. After culture for 48 hours, medium was changed to the selective growth medium supplemented with 650 μg/mL geneticin (MP Biomedicals, 0215878291) and cells were cultured for two weeks. Screened cells were used as MACF1 overexpression MC3T3‐E1 cells and BMSCs.

Stable MACF1 knockdown MC3T3‐E1 cell line was made by transfection of lentivirus vector carrying shRNA targeting murine MACF1 (NM_001199136.1) or its scramble control as described previously.^23^

### 2.8 Alkaline phosphatase staining and Alizarin Red staining

ALP staining and Alizarin Red staining were performed to determine osteogenic differentiation. Alkaline phosphatase (ALP) of MC3T3‐E1 cells and BMSCs was stained using BCIP/NBT Alkaline Phosphatase Color Development Kit (Beyotime Biotechnology, C3206, Shanghai, China) according to the manufacturer's instruction. Briefly, cells were washed with PBS (pH 7.4) and fixed in 10% buffered formaldehyde. The formaldehyde was washed by PBS, and then, cells were stained using 5‐bromo‐4‐chloro‐3‐indolyl phosphate (BCIP)/nitro blue tetrazolium (NBT) solution. The staining reaction was stopped by washing with tap water, and the cell staining was imaged by CanoScan 9000F Mark II Scanner (Canon, Tokyo, Japan).

For Alizarin Red staining, cells were cultured in osteogenic medium and the medium was replaced every 2 days. The staining was carried out on day 22 for MC3T3‐E1 cells and day 26 for BMSCs, and cells were washed with distilled water and then stained in 0.5% Alizarin Red S (pH 4.0; Sigma, A5533) for 30 mins. After immersed by tap water for 30 mins, the plates were dried and scanned with CanoScan 9000F Mark II Scanner.

### 2.9 Bone histomorphometric analyses

Bone histomorphometric analysis was performed to investigate the effect of ageing on bone formation and the rescue effect of MACF1 on osteoporosis. Collected calvarias were fixed with 4% paraformaldehyde and cut along the coronal plane 1.5 mm in front of the lambdoidal suture. Thickness of calvaria was measured by 3D Super Depth Digital Microscope (Hirox).

To measure mineral appositional rate, double calcein labelling was performed by intraperitoneal injection with calcein green (30mg/kg bodyweight; Sigma, C0875) in the time sequence of 11 and 3 days before euthanasia for specimen collection. Collected calvaria and femur samples were fixed with 4% paraformaldehyde, dehydrated by 50% sucrose and embedded in OCT (Leica, 14020108926, Wetzlar, Germany). Transverse cryosections (5 μm in thickness) were made by a freezing microtome (Leica, CM1100), and slides were examined with a fluorescent microscope (Nikon 80i). Bone dynamic histomorphometric analyses for mineral apposition rate (Shannon *et al*) were performed with image analysis software (ImageJ; National Institutes of Health, Bethesda, MD).^22^

TRAP staining was performed to measure femoral bone resorption, with paraffin sections and an Acid Phosphatase, Leukocyte (TRAP) Kit (Sigma‐Aldrich) according to the manufacturer's instructions, and the number of TRAP^+^ cells was counted using Image‐Pro Plus 6.0 software. Serum parathormone (PTH), β‐C‐telopeptides of type 1 collagen (β‐CTX) and type 1 amino‐terminal pro‐peptide (PINP) levels were measured by electrochemiluminescence.

### 2.10 Micro‐CT analysis

To investigate the rescue effect of MACF1 on ageing‐related osteoporosis, mice distal femoral was scanned by the micro‐CT system (version 6.5; viva CT40, Scando Medical, Switzerland). The femur was fixed by 70% ethanol and analysed by micro‐CT. Images of femurs were reconstructed and calibrated at the isotropic voxel size of 10.5 μm, respectively (70 kVp, 114 μA, 200‐ms integration time, 260 thresholds, 1200 mg HA/cm3). Using the Scanco evaluation software, regions of interest (ROIs) were defined for trabecular parameters. The entire femora were reoriented with the mid‐diaphysis parallel to the z‐axis, and bone length was measured as the distance between the most proximal and distal transverse plans containing the femur. Starting from the most proximal aspect of the growth plate, the trabecular region on 100 consecutive slices was selected. The trabeculae were analysed by manually contouring excluding the cortical bone for three‐dimensional reconstruction (sigma = 1.2, supports = 2 and threshold = 200) to calculate the following trabecular parameters including bone mineral density (BMD), bone volume to tissue volume (BV/TV), trabecular number (Tb.N), trabecular separation (Tb. Sp), bone mineral content (BMC) for trabecular microarchitecture and BMD for cortical microarchitecture.

### 2.11 Luciferase reporter assay

To analyse the potential function of MACF1 in regulating HES1 activity in osteoblastic cells, HES1 luciferase reporter plasmid was constructed by inserting 8 repeats of HES1 motif sequence to the promoter region of NanoLuc luciferase gene sequence in PNL1.1 plasmid (N1351; Promega). MACF1 overexpression or MACF1 knockdown MC3T3‐E1 cells and primary BMSCs were seeded on a 6‐well plate at 1 × 10^6^ cells/well, and HES1 luciferase reporter plasmid was transfected into MC3T3‐E1 cells using Engreen Entranster^TM^‐H4000 Reagent (Engreen, 4000‐6), with blank NanoLuc luciferase plasmid as normal control. A luciferase reporter assay was performed with the Nano‐Glo® Luciferase Assay System (Promega, N1120) according to the manufacturer's instruction. Briefly, 100μL cell culture medium was collected into a micro‐plate, and then, 100 μL diluted Nano‐Glo® Luciferase Assay Substrate was added. Nanoluc luciferase luminescent signals were quantified by a microplate reader (Synergy, USA) with 460 nm, and each value from the NanoLuc luciferase constructs was normalized by a normal control.

### 2.12 Therapeutic effect of MACF1 ageing and OVX mice

To investigate rescue effect of MACF1 to ageing‐related osteoporosis, forty‐two 21‐month‐old mice were randomly divided into three groups (21‐month, 21‐month+P‐C1 and 21‐month+P‐MACF1). In the 21‐month+P‐MACF1 group, mice received periosteal injection into medullary cavity of femur with PEGFP‐C1A‐ACF7 plasmid formulated with Entranster^TM^ In Vivo Transfection Reagent (Engreen Biosystem Co. Ltd., 18668‐11‐2, Beijing, China) at the dosage of 40 µL according to the manufacturer's instructions. In the 21‐month+P‐C1 group, mice were injected with PEGFP‐C1A plasmid with the same condition. In the 21‐month group, mice were given no treatment. The injection was performed at day 1, day 15 and day 29. All mice received the same standard diet during the experimental period. Mice were killed 48 days after the first injection, and femurs were collected. The femurs from mice were processed for BMSC isolation (n = 4/group), micro‐CT (n = 5/group) and histomorphometric analyses (n = 5/group), respectively.^17^

Ovariectomized (OVX) mice were constructed to investigate the rescue effect of MACF1 to post–menopausal‐related osteoporosis, 40 OVX mice were randomly divided into five groups (baseline, OVX, Mock, P‐C1 and P‐MACF1). The transfection was performed 6, 8 and 10 days after OVX, respectively. In the P‐MACF1 group, mice were injected subcutaneously over the calvarial surface with PEGFP‐C1A‐ACF7 plasmid formulated with Entranster^TM^ In Vivo Transfection Reagent (Engreen Biosystem Co. Ltd., 18668‐11‐2) twice per day, at the dosage of 40 µL according to the manufacturer's instructions. In the P‐C1 group, mice were injected with PEGFP‐C1A plasmid mixed with the same transfection reagent. In the mock group, mice received the same volume of normal saline mixed with transfection reagent alone. In the baseline and OVX group, mice were given no treatment. All mice received the same standard diet during the experimental period. All mice of the baseline group and 3 mice from other groups were killed 15 days after OVX treatment. All other mice were killed 38 days after OVX, and calvarias were collected. For baseline group, mice were killed 10 days after OVX treatment. The posterior calvarias were processed for immunohistochemical staining (n = 8/group). Anterior calvarias were used for RT‐PCR (n = 3/group) and histomorphometric analyses (n = 5/group).^22^

### 2.13 Statistical analysis

All experiments were independently repeated at least three times with each done in triplicate. The statistical analyses of the data were performed with GraphPad Prism version 6.0 software (GraphPad Software Inc), and a Student t‐test was used. The data are presented as mean ± standard deviation (SD). *P* values <.05 were considered statistically significant for all comparisons.

## 3 RESULTS

### 3.1 Reduced MACF1 level is associated with the reduction in osteogenesis in elderly people

**FIGURE 1:**
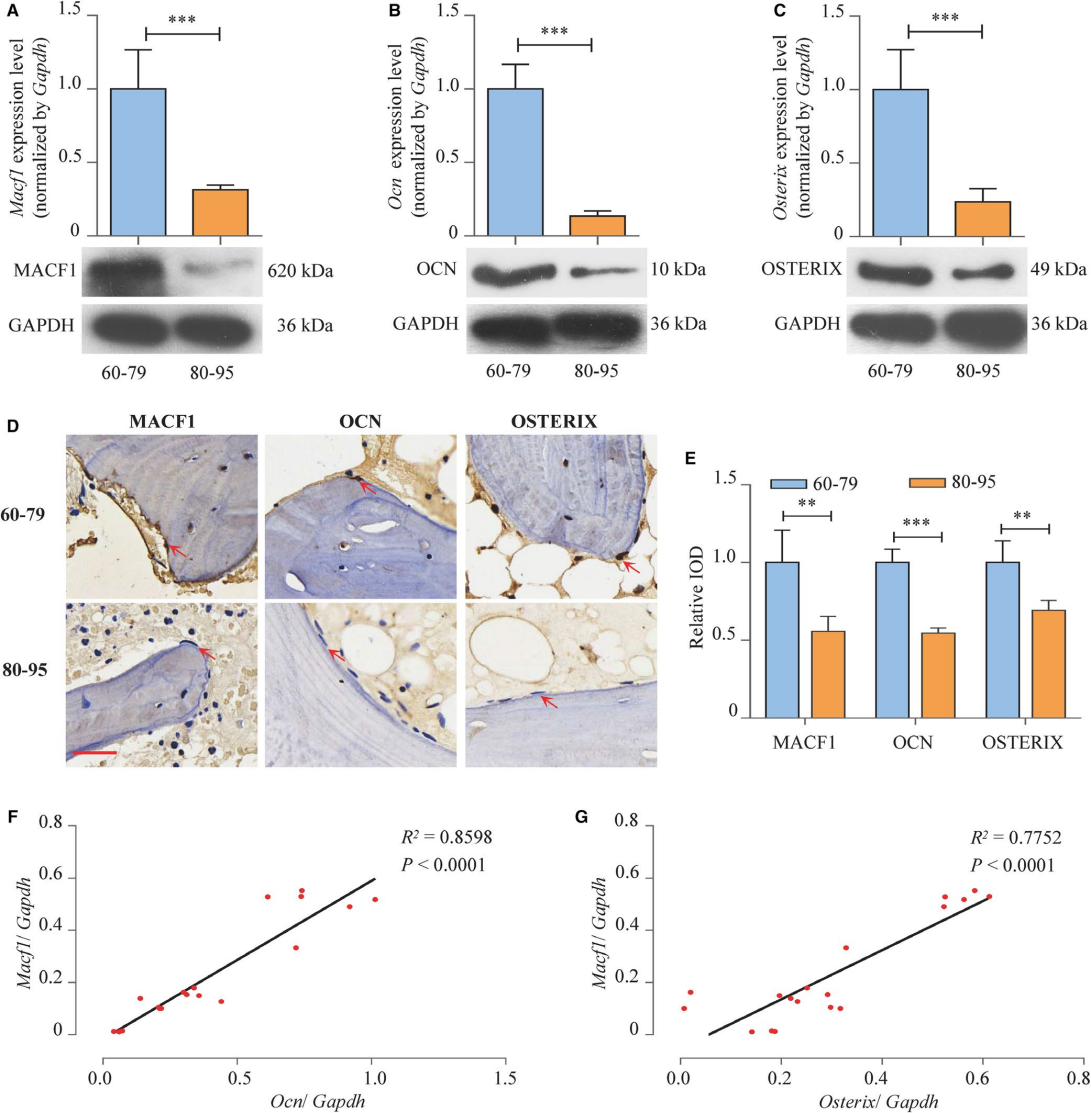
The decreased MACF1 level is associated with bone formation reduction in ageing‐related osteoporosis patient femur tissues. A‐C, Expression of MACF1, OCN and OSTERIX in femur tissues of ageing‐related osteoporosis patients with age of 60‐79 and 80‐95, respectively, as detected by RT‐PCR (up) and Western blot (below) (mean ± SD, ***P < .001). D, Expression of MACF1, OCN and OSTERIX in femur tissues of ageing‐related osteoporosis patients with age of 60‐79 and 80‐95, as detected by immunohistochemical staining. Scale bar: 50 μm. E, Quantification of relative integrated optical density (IOD) values of MACF1, OCN and OSTERIX immunostaining using Image‐Pro Plus 6.0 software (mean ± SD, **P < .01, ***P < .001). F‐G, Correlation analysis between *Macf1* levels and *Ocn* or *Oxterix* mRNA levels in femur tissues from ageing‐related osteoporosis patients, respectively, as detected by RT‐PCR

The expression level of MACF1 in femur tissues of patients with different ages was analysed. Average mRNA level of *Macf1* in female femur (80‐95 years old) was decreased by 68.5% (*P* < .001) compared with 60‐ to 79‐year‐old female patients. MACF1 protein level was also significantly decreased (Figure 1A). The above finding implied that the expression of MACF1 in weight‐bearing bones was decreased with age. The mRNA and protein expression levels of osteogenic marker *Ocn* (osteocalcin) and *Osterix* were also decreased in 80‐ to 95‐year elderly people (Figure 1B‐C). Immunohistochemical (IHC) staining of MACF1, OCN and OSTERIX also showed a significant decrease in the trabecular bone osteogenic cells of 80‐ to 95‐year‐old patients femur compared with the 60‐ to 79‐year‐old ones (Figure 1D‐E). In addition, the correlation analysis of different age patients showed that there was a positive relationship between *Macf1* expression and the expression of *Ocn* and *Osterix* (Figure 1F‐G). These data suggested that during the ageing process, declination of osteogenesis was strongly correlated with the decreased expression of MACF1.

### 3.2 Reduced MACF1 level is associated with the reduction in bone formation and osteogenic differentiation in ageing‐related osteoporosis mice

**FIGURE 2:**
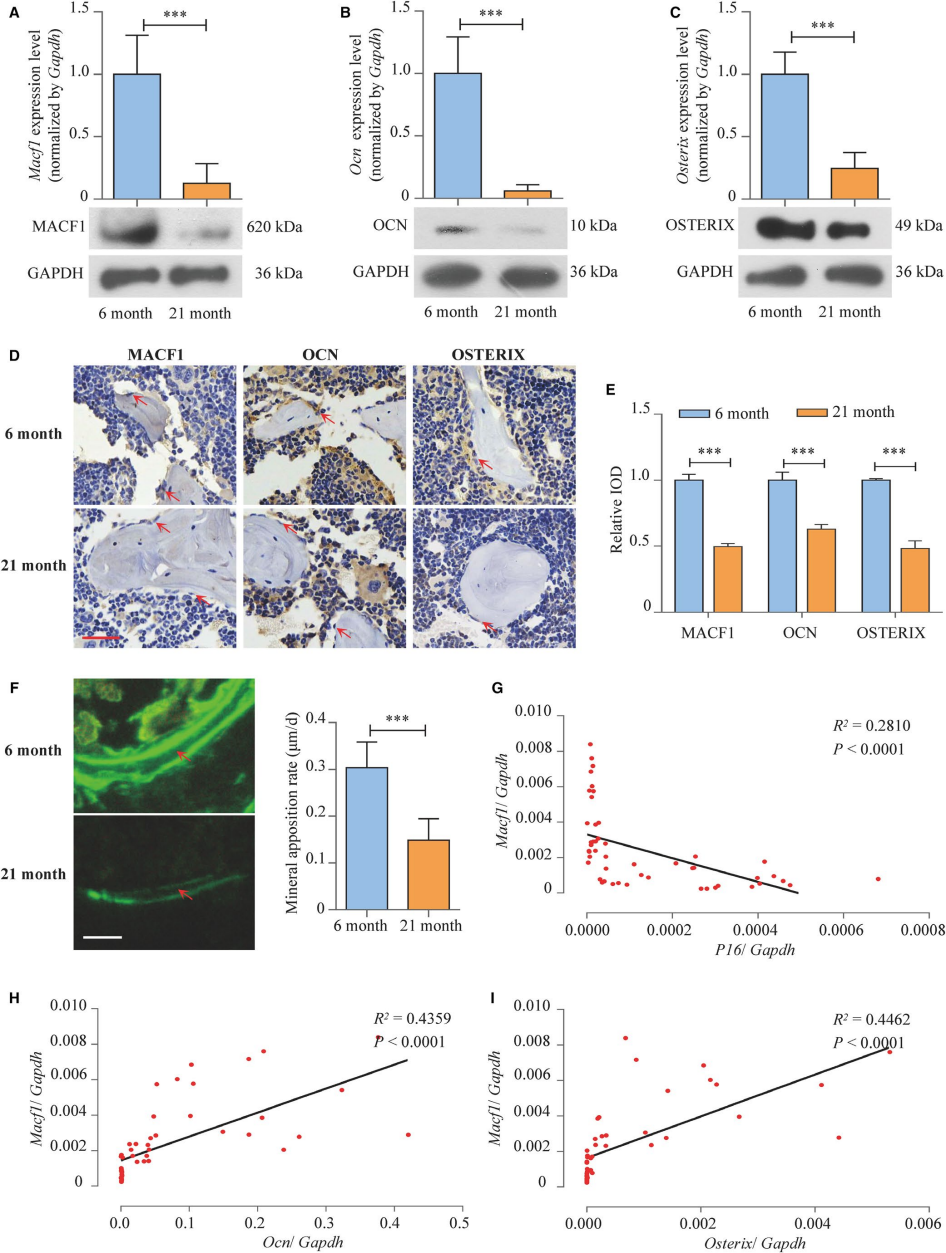
The decreased MACF1 level is associated with bone formation reduction in ageing mice femur tissues. A‐C, Expression of MACF1, OCN and OSTERIX in femur tissues of 6‐ and 21‐mo male C57BL/6 mice, respectively, as detected by RT‐PCR (up) and Western blot (below) (mean ± SD, ***P < .001). D, Expression of MACF1, OCN and OSTERIX in distal femur tissues of 6‐ and 21‐mo male C57BL/6 mice, as detected by immunohistochemical staining. Scale bar: 50 μm. E, Quantification of relative integrated optical density (IOD) values of MACF1, OCN and OSTERIX immunostaining using Image‐Pro Plus 6.0 software (mean ± SD, ***P < .001). F, Representative images showing distal femur mineral apposition rate of 6‐ and 21‐mo male C57BL/6 mice, as detected by double calcein labelling (mean ± SD, ***P < .001). Scale bar: 10 μm. G‐I, Correlation analysis between *Macf1* levels and *P16*, *Ocn* or *Oxterix* mRNA levels in femur tissues from C57BL/6 mice, respectively, as detected by RT‐PCR

We further detected the expression level of MACF1 in the femur of ageing mice. In the 21‐month ageing mice femur, *Macf1* mRNA level was decreased by 87.3% (*P* < .001) compared with 6‐month control mice, along with significant decrease in MACF1 protein (Figure 2A). The expression of OCN and OSTERIX was also decreased in ageing mice (Figure 2B‐C). Results detected by immunohistochemical staining showed that MACF1, OCN and OSTERIX were all decreased in the osteogenic cells of trabecular bone collected from ageing mice (Figure 2D‐E). Meanwhile, mineral apposition rate (MAR, an assessment of bone formation) in ageing mice was significantly lower, as compared to control (Figure 2F), which convinced that in ageing mice, MACF1 expression was decreased along with the reduction in osteogenic marker levels and bone formation.

Correlation analysis in different ages of C57BL/6 mice showed *Macf1* expression was negatively correlated with *P16*, which is a biomarker of ageing^24^ (Figure 2G). The *Macf1* expression was positively correlated with the expression of *Ocn* and *Osterix* (Figure 2H‐I), which was similar to the trend of ageing human.

**FIGURE 3:**
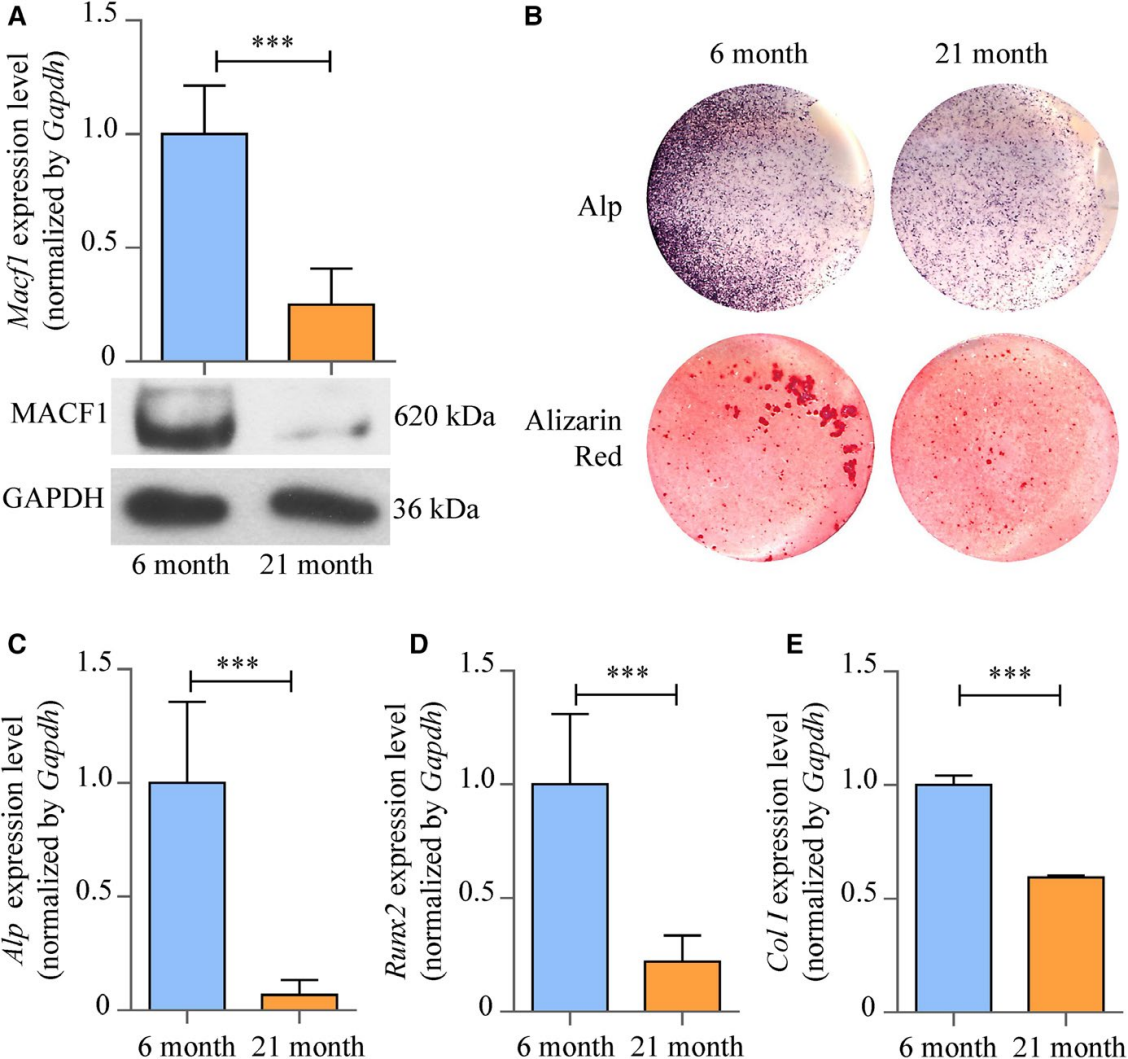
The decreased MACF1 level is associated with decreased osteogenic differentiation in bone marrow mesenchymal stem cells (BMSCs) isolated from ageing mice. A, Expression of MACF1 in BMSCs of 6‐ and 21‐mo male C57BL/6 mice, as detected by RT‐PCR (up) and Western blot (below) (mean ± SD, ***P < .001). B, Alp and Alizarin Red staining of BMSCs of 6‐ and 21‐mo male C57BL/6 mice, as detected by Alp staining and Alizarin Red staining. Alp: results of Alp staining. Alizarin Red: results of Alizarin Red staining. C‐E, Expression of *Alp*, *Runx2* and *Col I* in BMSCs of 6‐ and 21‐mo male C57BL/6 mice, respectively, as detected by RT‐PCR (mean ± SD, ***P < .001)

Bone marrow mesenchymal stem cells (BMSCs) were isolated from ageing mice femur, and MACF1 mRNA and protein level in 21‐month mice BMSCs was significantly lower compared with that in 6‐month mice (Figure 3A), which exhibited the same tendency as that in human and mice bone tissue. Moreover, ALP‐positive blue‐violet complexes and Alizarin Red–stained mineralized nodules were significantly decreased in ageing mice BMSCs (Figure 3B), and the expression of osteogenic marker gene *Alp* (alkaline phosphatase), *Runx2* (runt‐related transcription factor 2) and *Col I* (collagen type I) was also decreased by 93.2% (*P* < .001), 77.9% (*P* < .001) and 40.4% (*P* < .001), respectively (Figure 3C‐E). All these results proved that MACF1 might act as an important regulator of ageing, and decreased MACF1 expression is strongly associated with the reduction in osteogenic differentiation and bone formation.

### 3.3 MACF1 promotes bone formation by inhibiting the activities of HES1 in ageing‐related osteoporosis

**FIGURE 4:**
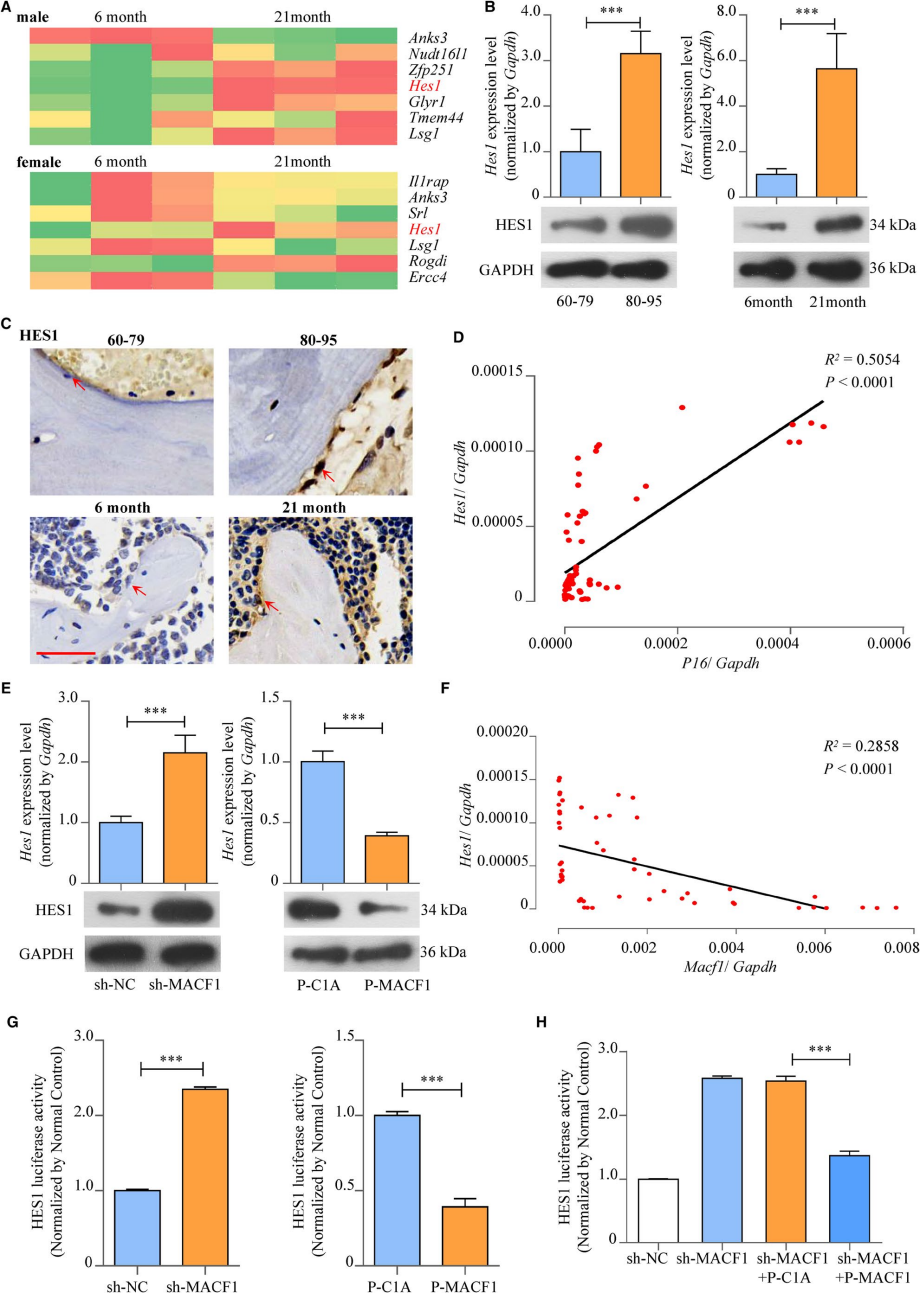
MACF1 promoted bone formation by inhibiting the activities of HES1 in ageing‐related osteoporosis. A, Selected area of RNA‐seq heat map for femur tissues of 6‐ and 21‐mo male (up) and female (below) C57BL/6 mice. B, Expression of HES1 in femur tissues of ageing‐related osteoporosis patients (left) and ageing C57BL/6 mice (right), as detected by RT‐PCR (up) and Western blot (below) (mean ± SD, ***P < .001). C, Expression of HES1 in femur tissues of ageing‐related osteoporosis patients (up) and ageing C57BL/6 mice (below), as detected by immunohistochemical staining. Scale bar: 50 μm. D, Correlation analysis between *Hes1* levels and *P16* mRNA levels in femur tissues from C57BL/6 mice, as detected by RT‐PCR. E, Expression of HES1 in MACF1 overexpression MC3T3‐E1 cells (left) and MACF1 knockdown MC3T3‐E1 cells (right), as detected by RT‐PCR (up) and Western blot (below) (mean ± SD, ***P < .001). F, Correlation analysis between *Macf1* levels and *Hes1* mRNA levels in femur tissues from C57BL/6 mice, as detected by RT‐PCR. G, Activities of HES1 in MACF1 overexpression MC3T3‐E1 cells (left) and MACF1 knockdown MC3T3‐E1 cells (right), as detected by luciferase reporter assay (mean ± SD, ***P < .001). H, Rescuing effect of MACF1 overexpression plasmid on HES1 activities of MACF1 knockdown MC3T3‐E1 osteoblastic cells, as detected by luciferase reporter assay (mean ±SD, ***P < .001)

The mechanism of how MACF1 regulated bone formation was further explored. We established RNA‐seq for femur tissues collected from both male and female ageing mice. We found the expression levels of *Hes1* were significantly increased in 21‐month ageing mice compared with 6‐month control (Figure 4A). The results were further confirmed by RT‐PCR, Western blot and immunohistochemical staining in ageing human and mice femur tissues (Figure 4B‐C, Figure S1). Moreover, in femurs of different ages of C57BL/6 mice, expression level of *Hes1* was positively correlated with *P16* (Figure 4D) convincing that HES1 expression was increased with the ageing process.

We then investigated the regulation effect of MACF1 on HES1. In MACF1 knockdown MC3T3‐E1 osteoblast, mRNA expression level of *Hes1* was enhanced by 115.1% (*P* < .001), and the protein level of HES1 was also significantly elevated. Although *Hes1* mRNA expression level presented a 60.9% decrease in MACF1 overexpression osteoblasts (*P* < .001), with a similar tendency in HES1 protein level (Figure 4E), HES1 activity was enhanced by 135% (*P* < .001) in MACF1 knockdown osteoblasts and was decreased by 60.8% (*P* < .001) in MACF1 overexpression osteoblasts (Figure 4G). Correlation analysis in different ages of human and C57BL/6 mice revealed *Hes1* expression was negatively related to *Macf1* (Figure S2, Figure 4F).

We further investigated the rescue effect of MACF1 overexpression plasmid to HES1 expression and activities. The transfection of MACF1 overexpression plasmid PEGFP‐C1A‐ACF7 significantly reduced HES1 mRNA expression and activity of MACF1 knockdown osteoblasts (Figure S3A, Figure 4H). Moreover, MACF1 overexpression plasmid also down‐regulated expression level of *Hes1* in 21‐month ageing mice BMSCs (Figure S3B). All these results implied that MACF1 inhibited the expression level and activities of HES1, an osteogenic inhibiting factor in ageing‐related osteoporosis.

### 3.4 Rescue effects of MACF1 on ageing‐related osteoporosis

**FIGURE 5:**
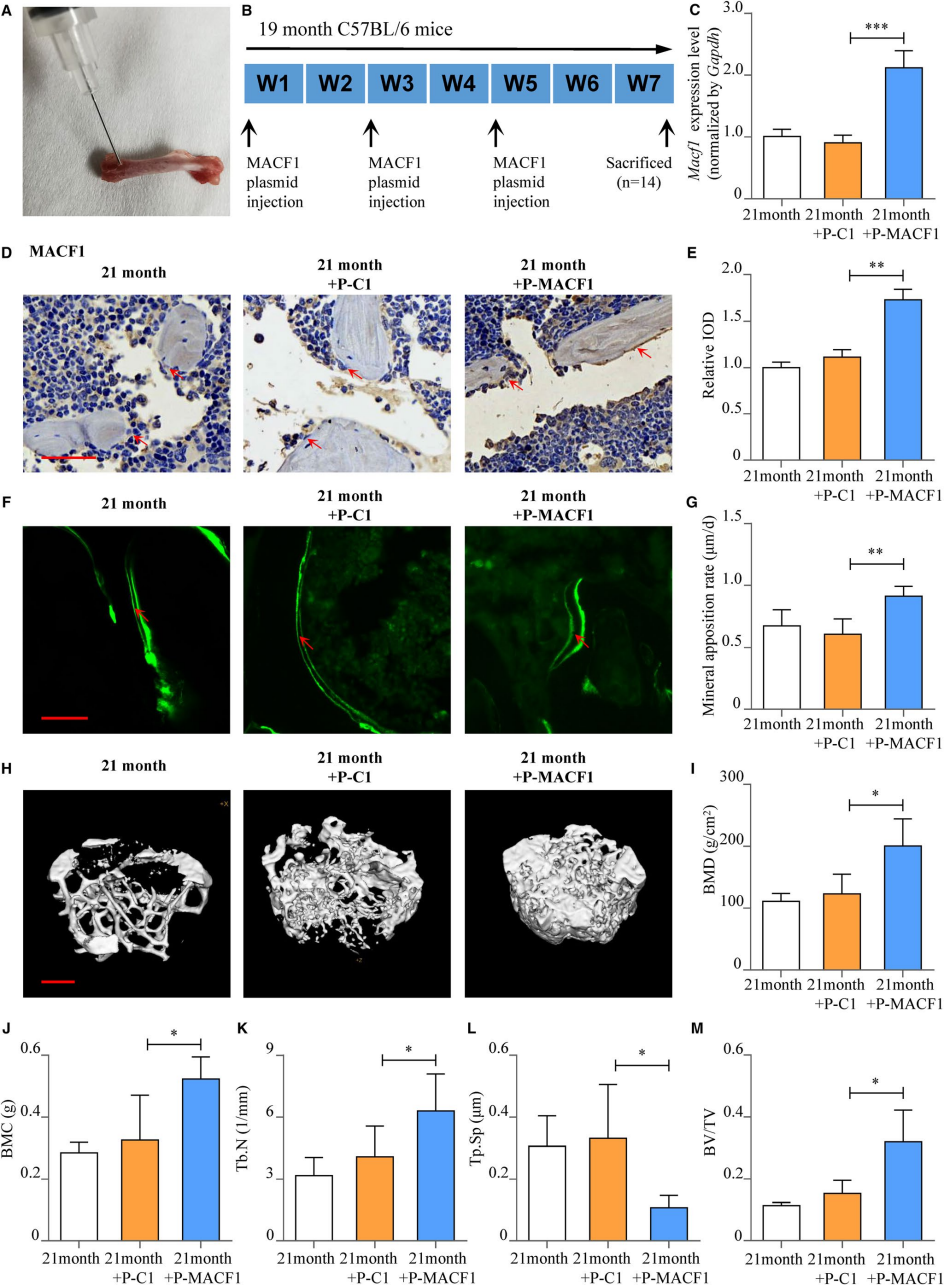
Rescue effect of MACF1 on senile osteoporosis. A, Puncture position of periosteal injection. B, Schematic graph showing experimental design. C, *Macf1* expression levels of ageing C57BL/6 mice BMSCs after MACF1 overexpression plasmid treatment, as detected by RT‐PCR (mean ± SD, ***P < .001). 21 mo: 21‐mo control group. 21 mo+P‐C1: blank plasmid–treated group. 21 mo+P‐MACF1: MACF1 overexpression plasmid‐treated group. D, Expression of MACF1 in femur tissues of ageing C57BL/6 mice distal femur after MACF1 overexpression plasmid treatment, as detected by immunohistochemical staining. Scale bar: 50 μm. E, Quantification of relative integrated optical density (IOD) values of MACF1 immunostaining using Image‐Pro Plus 6.0 software (mean ± SD, **P < .01). F, Representative images showing femoral mineral apposition rate of ageing C57BL/6 mice BMSCs after MACF1 overexpression plasmid treatment, as detected by double calcein labelling. Scale bar: 10 μm. G, Femoral mineral apposition rates of ageing C57BL/6 mice BMSCs after MACF1 overexpression plasmid treatment (mean ± SD, **P < .01). H, Representative images showing femoral trabecular microarchitecture of ageing C57BL/6 mice BMSCs after MACF1 overexpression plasmid treatment, as detected by micro‐CT. Scale bar: 500 μm. I‐M, Trabecular bone mineral density (BMD), bone mineral content (BMC), trabecular number (Tb.N), trabecular separation (Tb. Sp) and bone volume to tissue volume (BV/TV) of ageing C57BL/6 mice distal femur after MACF1 overexpression plasmid treatment, as detected by micro‐CT (mean ± SD, *P < .05)

For further evaluating the effects and mechanism of MACF1 on ageing‐related osteoporosis, 21‐month ageing C57BL/6 mice received a periosteal injection into medullary cavity of femur with MACF1 overexpression plasmid and transfection reagent^25^, ^26^, ^27^ (Figure 5A‐B). *Macf1* expression level in the BMSCs of MACF1 overexpression plasmid‐injected mice was enhanced by 133.3% (*P* < .001), compared to mice treated with control plasmid (Figure 5C). Similar tendency was shown in immunohistochemical staining results of the trabecular bone osteogenic cells (Figure 5D‐E). All of these results proved that periosteal injection of MACF1 overexpression plasmid successfully increased MACF1 expression in osteogenic cells of ageing mice. Osteogenic markers OCN and OSTERIX were enhanced by MACF1 in osteogenic cells of trabecular bone (Figure S4). The mineral apposition rate was enhanced by 50.8% (*P* < .01) in MACF1‐treated mice compared with control (Figure 5F‐G). Meanwhile, micro‐CT results showed that trabecular bone mineral density (BMD), bone mineral content (BMC), bone volume to tissue volume (BV/TV) and trabecular number (Tb.N) for trabecular bone were significantly enhanced by MACF1 in ageing mice, whereas trabecular separation (Tb. Sp) was significantly decreased (Figure 5H‐M). Yet, the bone mineral density in cortical bone showed no difference (Figure S5). Moreover, femoral TRAP staining and serum parathormone (PTH), and β‐C‐telopeptides of type 1 collagen (β‐CTX) level showed no significant differences in MACF1‐treated ageing mice and revealed the treatment of MACF1 showed no effect on femoral bone resorption (Figure S6A‐D). Serum level of type 1 amino‐terminal pro‐peptide (PINP) was enhanced and proved the rescue effect of MACF1 on bone formation (Figure S6E). All of these results proved the rescue effect of MACF1 on bone formation and trabecular microarchitecture in ageing mice.

**FIGURE 6:**
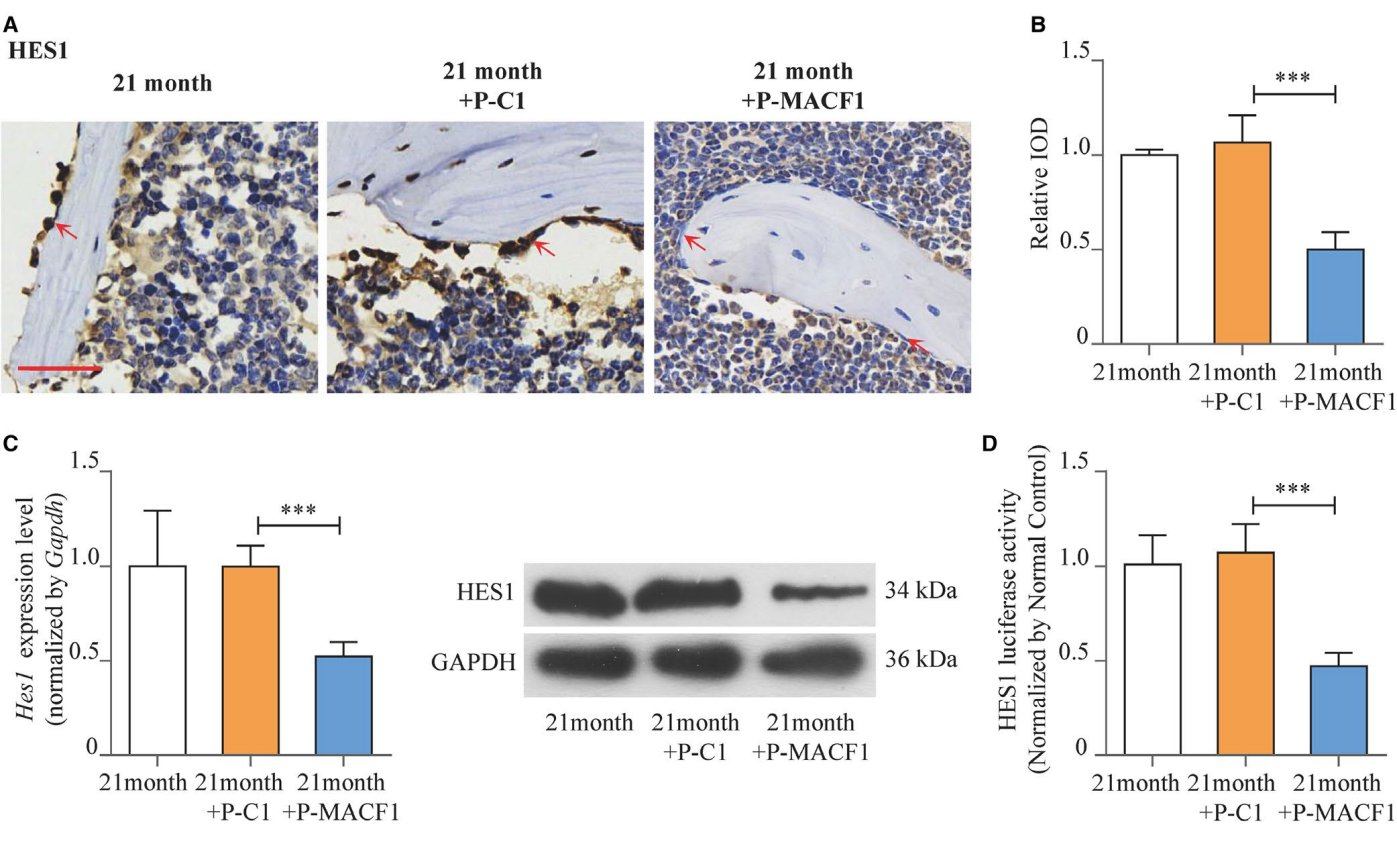
Rescue effect of MACF1 on HES1 expression and activity of ageing mice. A, Expression of HES1 in femur tissues of ageing C57BL/6 mice distal femur after MACF1 overexpression plasmid treatment, as detected by immunohistochemical staining. Scale bar: 50 μm. 21 mo: 21‐mo control group. 21 mo+P‐C1: blank plasmid–treated group. 21 mo+P‐MACF1: MACF1 overexpression plasmid‐treated group. B, Quantification of relative integrated optical density (IOD) values of HES1 immunostaining using Image‐Pro Plus 6.0 software (mean ± SD, **P < .01). C, Expression of HES1 in BMSCs of ageing C57BL/6 mice distal femur after MACF1 overexpression plasmid treatment, as detected by RT‐PCR (up) and Western blot (below) (mean ± SD, ***P < .001). D, Activities of HES1 in BMSCs of ageing C57BL/6 mice distal femur after MACF1 overexpression plasmid treatment, as detected by luciferase reporter assay (mean ± SD, ***P < .001)

Expression levels of HES1 in MACF1‐treated ageing mice were detected by immunohistochemical staining, and HES1 level in the trabecular bone osteogenic cells was reduced by MACF1 (Figure 6A‐B). In MACF1‐treated mice BMSCs, HES1 mRNA and protein levels were both significantly decreased, as compared with normal ageing mice and control plasmid–treated mice (Figure 6C). Moreover, the activity of HES1 in mice BMSCs also presented a 52.8% (*P* < .001) decrease after MACF1 treatment (Figure 6D). The results implied that MACF1 may relieve ageing‐related osteoporosis through inhibition of HES1.

### 3.5 Rescue effects of MACF1 on post‐menopausal osteoporosis

**FIGURE 7:**
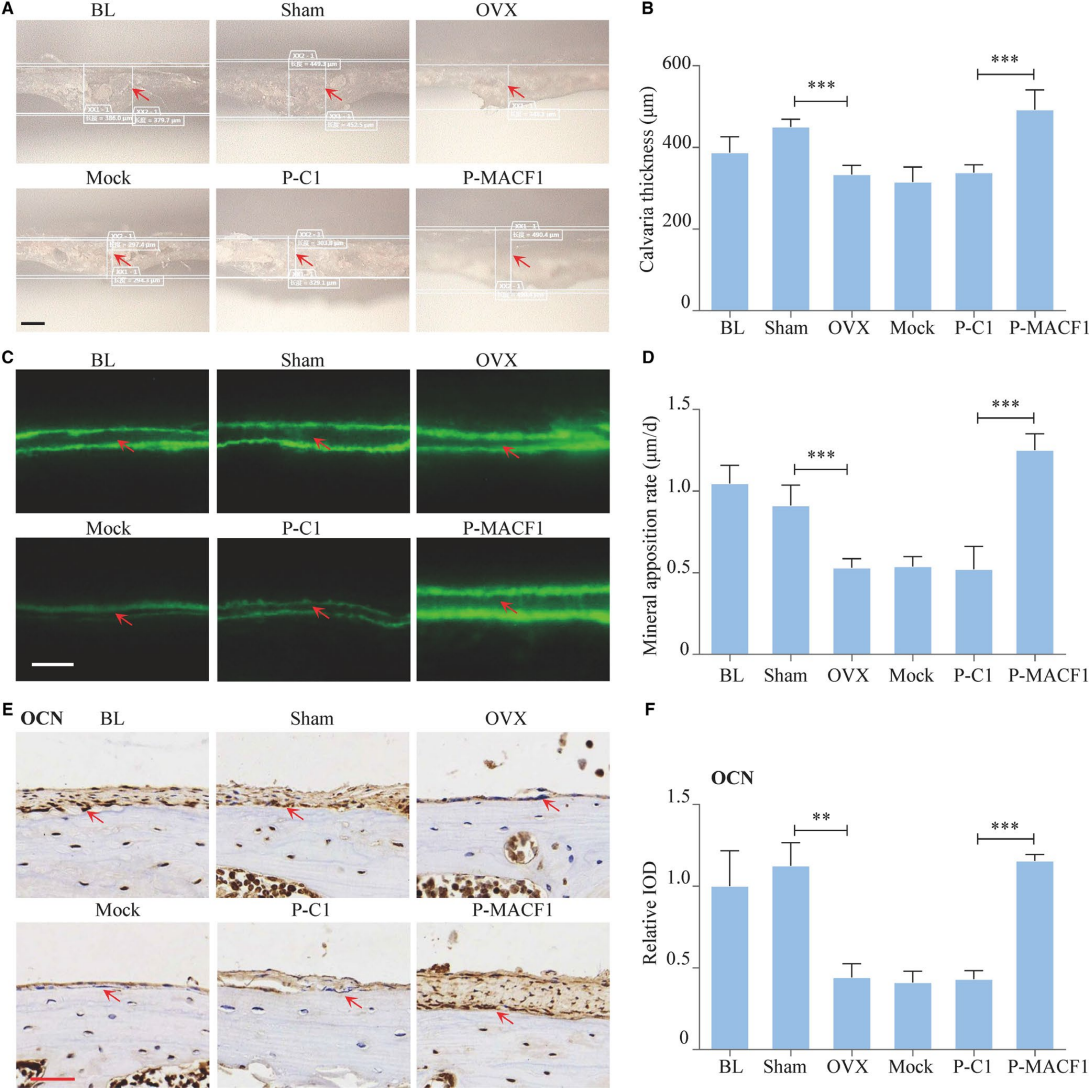
Rescue effect of MACF1 on post‐menopausal osteoporosis. A, Calvaria morphologies of C57BL/6 mice after OVX treatment and MACF1 overexpression plasmid transfection, as detected by Super Depth Digital Microscope. BL (Baseline): kill before MACF1 overexpression plasmid treatment. Sham: sham OVX operation group. OVX: OVX group. Mock: transfection reagent control group. P‐C1: blank plasmid–treated group. P‐MACF1: MACF1 overexpression plasmid‐treated group. Scale bar: 200 μm. B, Calvaria thickness of C57BL/6 mice after OVX treatment and MACF1 overexpression plasmid transfection (mean ± SD, ***P < .001). C, Representative images showing calvarial mineral apposition rate of C57BL/6 mice after OVX treatment and MACF1 overexpression plasmid transfection, as detected by double calcein labelling. Scale bar: 10 μm. D, Calvarial mineral apposition rates of C57BL/6 mice after OVX treatment and MACF1 overexpression plasmid transfection (mean ± SD, **P < .01). E, Expression of OCN in calvarial tissues of C57BL/6 mice after OVX treatment and MACF1 overexpression plasmid transfection, as detected by immunohistochemical staining. Scale bar: 50 μm. F, Quantification of relative integrated optical density (IOD) values of OCN immunostaining using Image‐Pro Plus 6.0 software (mean ± SD, ***P < .001)

We next investigated whether overexpression of MACF1 could rescue post‐menopausal osteoporosis. OVX mouse model was generated to simulate post‐menopausal osteoporosis. MACF1 overexpression plasmid was injected subcutaneously over the calvarial surface. The expression of MACF1 in calvarias was relatively unchanged by OVX surgery while was significantly up‐regulated upon plasmid transfection (Figure S7‐S8). Bone histomorphometric results showed that average calvarial thickness was decreased by ovariectomy and increased by MACF1 treatment (Figure 7A‐B). Meanwhile, the mineral apposition rate in the OVX mice was decreased by 41.8% (*P* < .001) and increased by 139.8% (*P* < .001) after MACF1 overexpression plasmid treatment (Figure 7C‐D). Immunohistochemistry staining results showed the protein levels of OCN were significantly down‐regulated after OVX surgery, while was up‐regulated by MACF1 (Figure 7E‐F). These data suggested that enhancing the expression level of MACF1 could promote bone formation and therefore rescue the minus consequence of menopause.

## 4 DISCUSSION

Ageing‐related osteoporosis is a prevalent bone disease in ageing population. The patients would have deteriorated bone microstructure, decreased bone mass and bone strength, suffering hunchback, muscle spasms, myasthenia, pain and increased risk of fracture.^1^ Along with the ageing of human population in recent years, the incidence of ageing‐related osteoporosis is elevated and had become an emerging threat to elderly population. Multiple factors could be the causes of ageing‐related osteoporosis, including genetics, hormone disorder, malnutrition, overuse of drugs and disuse.^3^ Among all reasons, the most essential cause of ageing‐related osteoporosis is the inhibition of differentiation of osteogenic cells during the process of ageing and post‐menopausal, which results in decreased bone formation and slows down the rate of bone reconstruction, and further causes imbalanced bone turnover and eventually lead to osteoporosis.

The differentiation of osteogenic cells can be impacted by many factors. MACF1 is a cytoskeletal protein that regulates actin and microtubule dynamics.^8^, ^9^, ^10^, ^11^, ^12^ It also plays a role in regulating intracellular transport.^14^, ^15^ Our previous studies have shown that MACF1 positively regulated osteoblast differentiation and bone formation. Qian has proved that MACF1 highly expressed in pre‐osteoblast cell line MC3T3‐E1.^28^ Hu constructed MACF1 knockdown MC3T3‐E1 cells, and proved MACF1 promotes osteoblast proliferation and differentiation.^16^, ^23^ Yin and Zhang adopted MACF1 overexpression plasmid and developed the technique to overexpress MACF1 within osteoblasts in vitro and in vivo, and they also found MACF1 overexpression would enhance osteoblast differentiation and bone formation.^17^, ^21^ Zhao and Qiu found that MACF1 regulated bone formation via SMAD7 and BMP2 through constructing MACF1 conditional knocked out mice in mesenchymal stem cells and osteoblasts, respectively.^18^, ^19^ However, as an important regulator for bone formation, few researchers have investigated the expression level of MACF1 during ageing process, and the relationships between MACF1 expression and ageing‐related osteoporosis still remain unclear. This study has revealed MACF1 expression was decreased during the ageing process, and the negative correlation between MACF1 and ageing biomarker gene cyclin‐dependent kinase inhibitor 2A (p16) was also found. Moreover, reduced MACF1 expression was associated with the reduction in osteogenic differentiation and bone formation as well (Figures 1, 2, 3). Through all these results, we have firstly proved that MACF1 act as an important regulator for ageing‐related osteoporosis.

Reports have shown that during the process of ageing, the expression of both Wnt/β‐catenin and NOTCH signalling pathways was impacted, and the differentiation of osteogenic cells was decreased, thus triggered the consecutive process of osteoporosis.^4^, ^5^, ^6^ In previous studies, MACF1 has been proved participating in Wnt/β‐catenin pathway and allowing β‐catenin to dissociate from the β‐catenin/AXIN/APC complex and thus transport to cell nucleus.^14^, ^21^, ^23^ We then diverted our target to NOTCH signalling pathway. HES1 functions as the downstream transcription factor of the canonical Notch signalling pathway. Reports have shown that HES1 inhibited osteoblast differentiation, and its expression level was enhanced during ageing‐related osteoporosis.^5^, ^6^, ^7^ In this study, we also proved that HES1 expression was increased in femur tissues of ageing human and mice. We have also found MACF1 negatively regulated HES1 expression and activities (Figure 4), which indicated a potential mechanism for MACF1 regulating ageing‐related osteoporosis. The internal mechanism of how MACF1 inhibits HES1 expression remains to be further studied.

Previous reports have demonstrated that the implementation of MACF1 overexpression plasmid would enhance bone formation of mice calvaria.^17^ Yet, the symptoms in ageing‐related osteoporosis mostly affect weight‐bearing bones, especially femur, lumbar vertebra and tibia.^29^, ^30^ In Li, Yuan and Zhao's studies, miRNAs or siRNAs targeting osteogenic genes were administrated into the bone marrow of mice femur by periosteal injection and enhanced bone formation.^25^, ^26^, ^27^ In this study, MACF1 overexpression plasmid was injected into medullary cavity of mice femur by the same technique, and the treatment had successfully inhibited HES1 level and enhanced ageing mice bone formation (Figures 5, 6), which firstly proved plasmids could be adopted in periosteal injection rescue, and also suggested that overexpression of MACF1 would be a potential therapeutic strategy for ageing‐related osteoporosis.

However, MACF1 was also proved enhancing the activities of osteoclast.^31^ The overexpression of MACF1 might also aggravate bone resorption of ageing mice. We have measured the bone resorption levels of MACF1‐treated ageing mice and found no significant differences. To clarify this conflict, we analysed the transfection efficiency of the large MACF1 overexpression plasmid (21kb) on osteoclast. Result showed the transfection efficiency was extremely low in both monocytic RAW264.7 cell line and extracted primary osteoclast, which may explain the reason why MACF1 overexpression plasmid treatment showed no effect on ageing mice bone resorption.

Moreover, the effect of MACF1 on post‐menopausal osteoporosis was also proved. MACF1 overexpression plasmid was injected subcutaneously over the calvarial surface.^22^ We found that MACF1 expression was not impacted by ovariectomy, but enhancing MACF1 expression would still rescue bone formation in post‐menopausal osteoporosis (Figure 7). The function of MACF1 in both osteoporosis models has proved the rescuing function of MACF1 on osteoporosis. However, the therapeutic technique with plasmid has low efficiency and high risk. So, we are establishing new methods to enhance MACF1 expression, such as regulating MACF1 enhancer or promoter by transcription factors of CRISPR‐SAM system in our further studies. Moreover, applying the vectors with aptamers targeting osteogenic cells would further enhance the efficiency and safety of this therapeutic technique.^32^

Through this study, we have discovered the relationship and mechanism between MACF1 and ageing‐related osteoporosis. MACF1 expression was reduced during the ageing process. The reduction in bone formation in ageing‐related osteoporosis was caused by decreased MACF1 level leading to disabled suppression of HES1 expression. Through these findings, we have proved that enhancing MACF1 expression would rescue ageing and post–menopausal‐related osteoporosis. This study has discovered a new molecular mechanism for ageing‐related osteoporosis and also provided new insights for therapeutic strategy of osteoporosis.

## CONFLICT OF INTEREST

No conflicts of interest.

## AUTHOR CONTRIBUTION

**Chong Yin:** Formal analysis (lead); Investigation (lead); Methodology (lead); Project administration (lead); Software (supporting); Validation (lead); Visualization (lead); Writing‐original draft (lead); Writing‐review & editing (equal). **Ye Tian:** Project administration (lead); Validation (lead); Visualization (lead); Writing‐review & editing (lead). **Lifang Hu:** Project administration (supporting); Supervision (supporting); Validation (supporting). **Yang Yu:** Funding acquisition (supporting); Project administration (supporting); Software (lead); Validation (equal); Writing‐review & editing (lead). **Zixiang Wu:** Investigation (equal); Methodology (equal); Validation (equal). **Yan Zhang:** Methodology (equal); Validation (supporting). **Xue Wang:** Investigation (supporting); Methodology (supporting); Validation (equal). **Zhi‐Ping Miao:** Funding acquisition (lead); Validation (equal); Writing‐review & editing (equal). **Ai‐Rong Qian:** Conceptualization (lead); Funding acquisition (lead); Project administration (lead); Resources (lead); Supervision (lead); Validation (lead); Visualization (lead); Writing‐review & editing (lead).

## Supporting information

Fig S1‐S8

## Data Availability

The data used to support the findings of this study are available from the corresponding author upon request.
