# Supplementary material for: MACF1 alleviates aging‐related osteoporosis via HES1
Source: J Cell Mol Med. 2021 Jun 15;25(13):6242–57. doi: 10.1111/jcmm.16579 (PMC8366449; doi:10.1111/jcmm.16579)
Supplement: Supplementary file 1 — Fig S1‐S8 [file JCMM-25-6242-s001.docx]

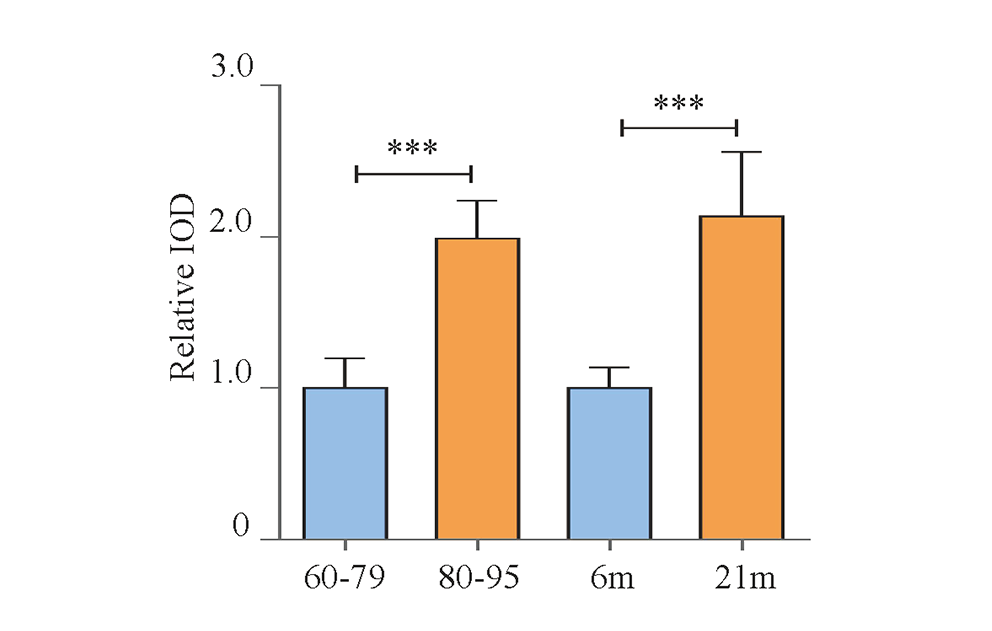


**Supplemental Figure 1.** Quantification of relative integrated optical density (IOD) values of HES1 immunostaining in femur tissues of aging-related osteoporosis patients and aging C57BL/6 mice using Image-Pro Plus 6.0 software (mean ± SD, ****P*<0.001).


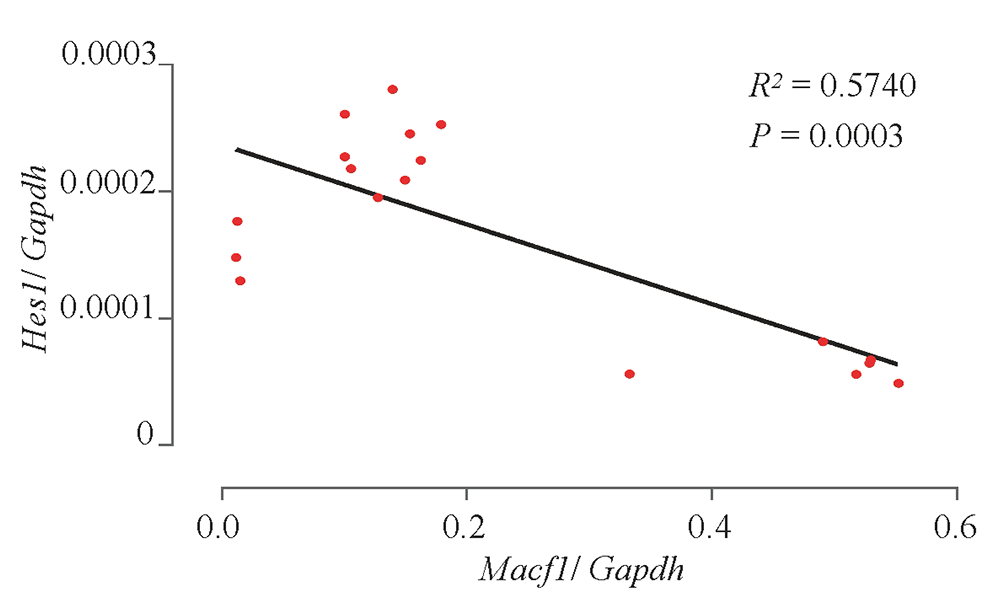


**Supplemental Figure 2.** Correlation analysis between *Macf1* levels and *Hes1* mRNA levels in femur tissues from aging-related osteoporosis patients, as detected by RT-PCR.


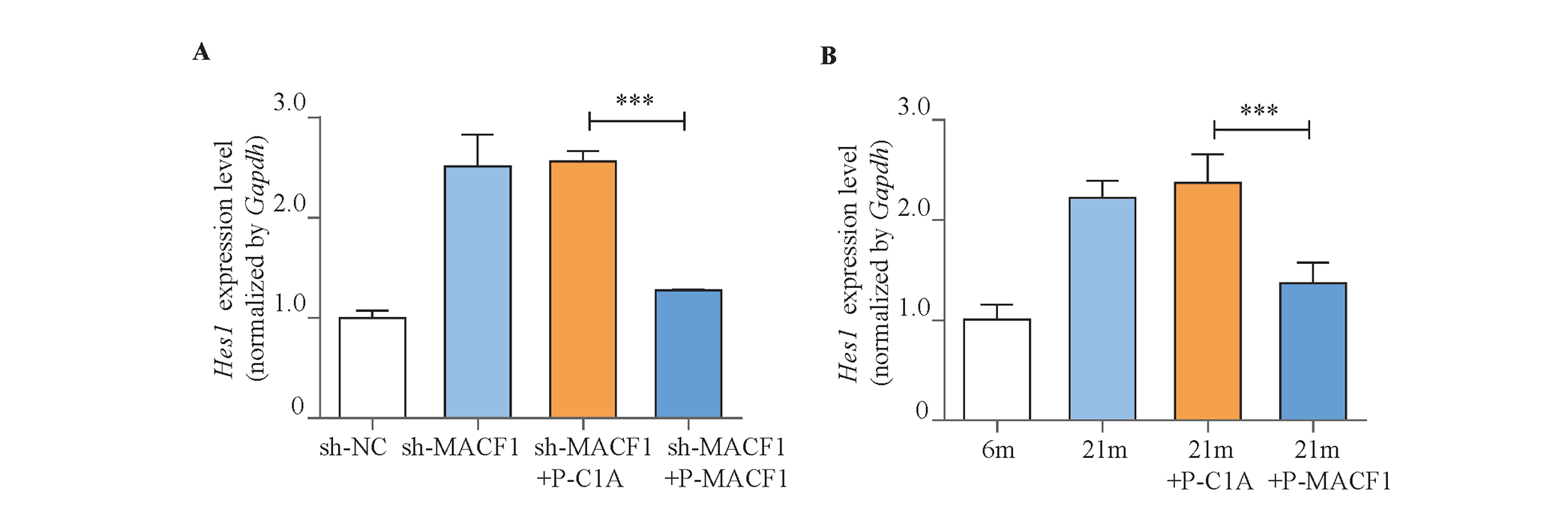


**Supplemental Figure 3. Rescuing effect of MACF1-overexpression plasmid on HES1 expression levels and activities**

**A.** Rescuing effect of MACF1-overexpression plasmid on HES1 expression levels of MACF1-knockdown MC3T3-E1 osteoblastic cells, as detected by luciferase reporter assay (mean ± SD, ****P*<0.001).

**B.** Rescuing effect of MACF1-overexpression plasmid on HES1 activities of aging mice BMSCs, as detected by luciferase reporter assay (mean ± SD, ****P*<0.001).


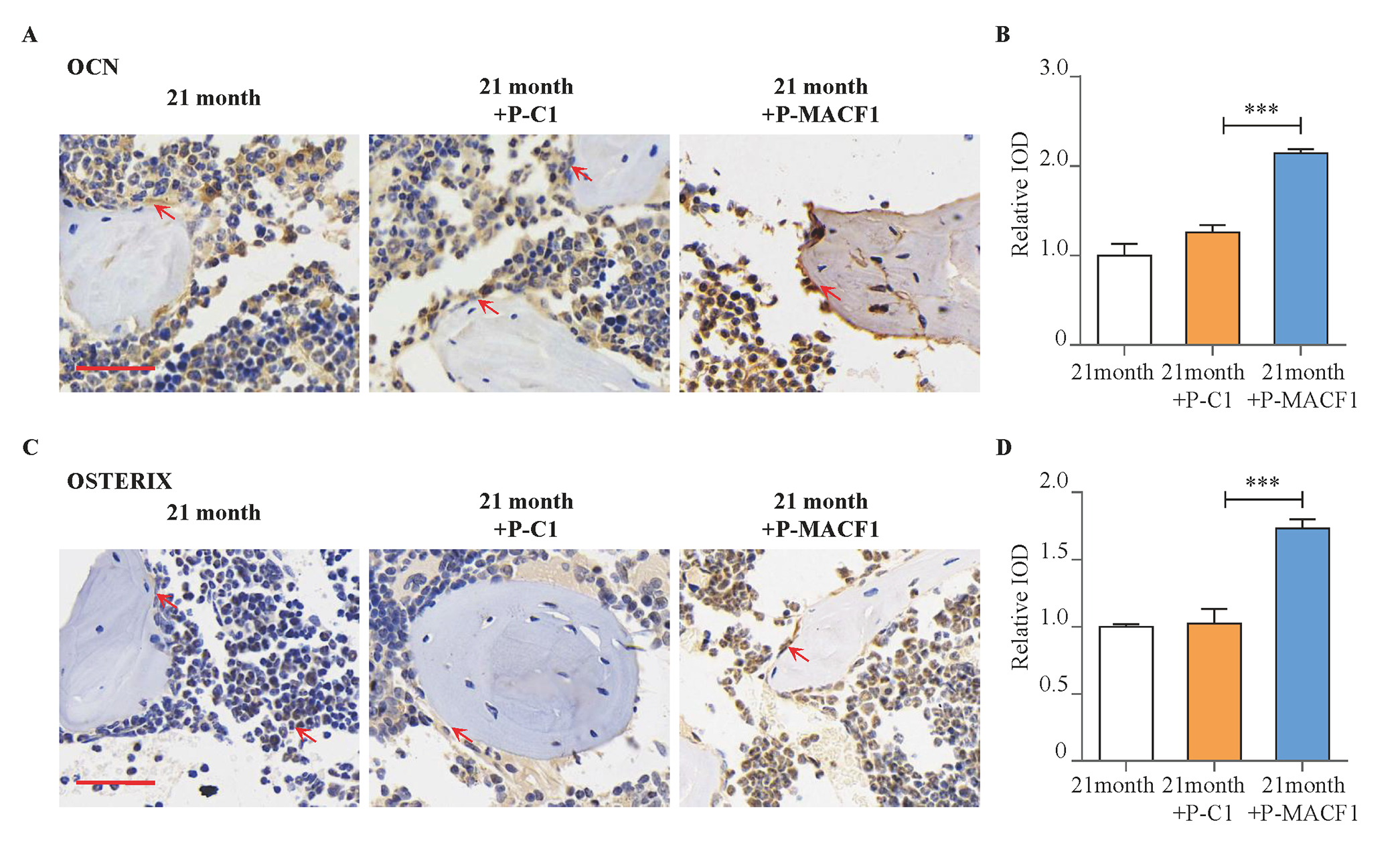


**Supplemental Figure 4. Effect of MACF1 on aging mice osteogenic markers**

**A.** Expression of OCN in femur tissues of aging C57BL/6 mice distal femur after MACF1 over-expression plasmid treatment, as detected by immunohistochemical staining. Scale bar: 50μm. 21month: 21-month control group. 21month+P-C1: blank plasmid treated group. 21month+P-MACF1: MACF1 over-expression plasmid treated group.

**B.** Quantification of relative integrated optical density (IOD) values of OCN immunostaining using Image-Pro Plus 6.0 software (mean ± SD, ****P*<0.001).

**C.** Expression of OSTERIX in femur tissues of aging C57BL/6 mice distal femur after MACF1 over-expression plasmid treatment, as detected by immunohistochemical staining. Scale bar: 50μm.

**D.** Quantification of relative integrated optical density (IOD) values of OSTERIX immunostaining using Image-Pro Plus 6.0 software (mean ± SD, ****P*<0.001).


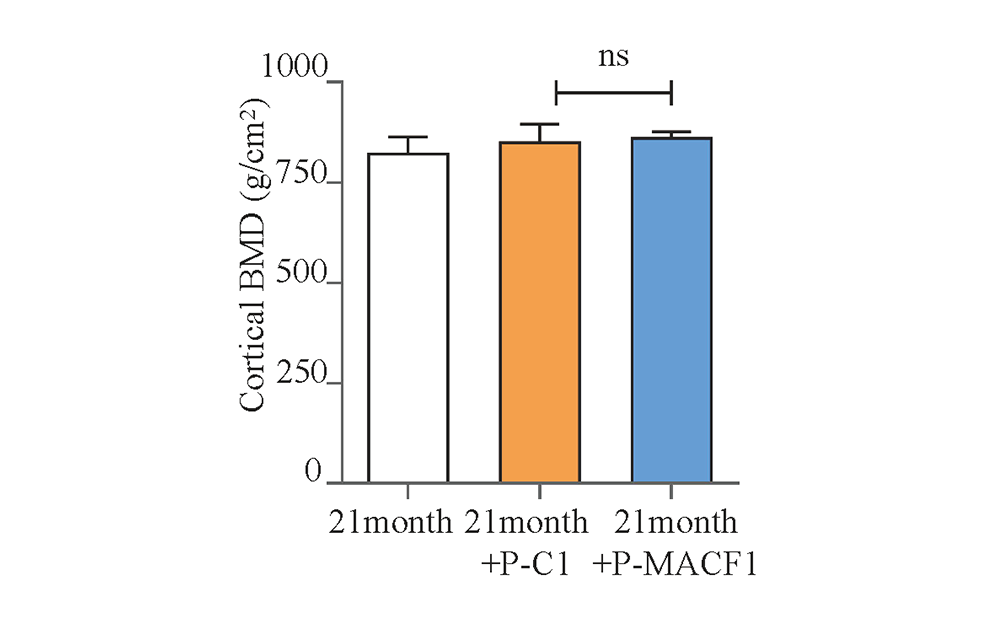


**Supplemental Figure 5. Effect of MACF1 on aging mice cortical bone**

Cortical bone mineral density (BMD) of aging C57BL/6 mice distal femur after MACF1 over-expression plasmid treatment, as detected by micro CT (mean ± SD). 21month: 21-month control group. 21month+P-C1: blank plasmid treated group. 21month+P-MACF1: MACF1 over-expression plasmid treated group.


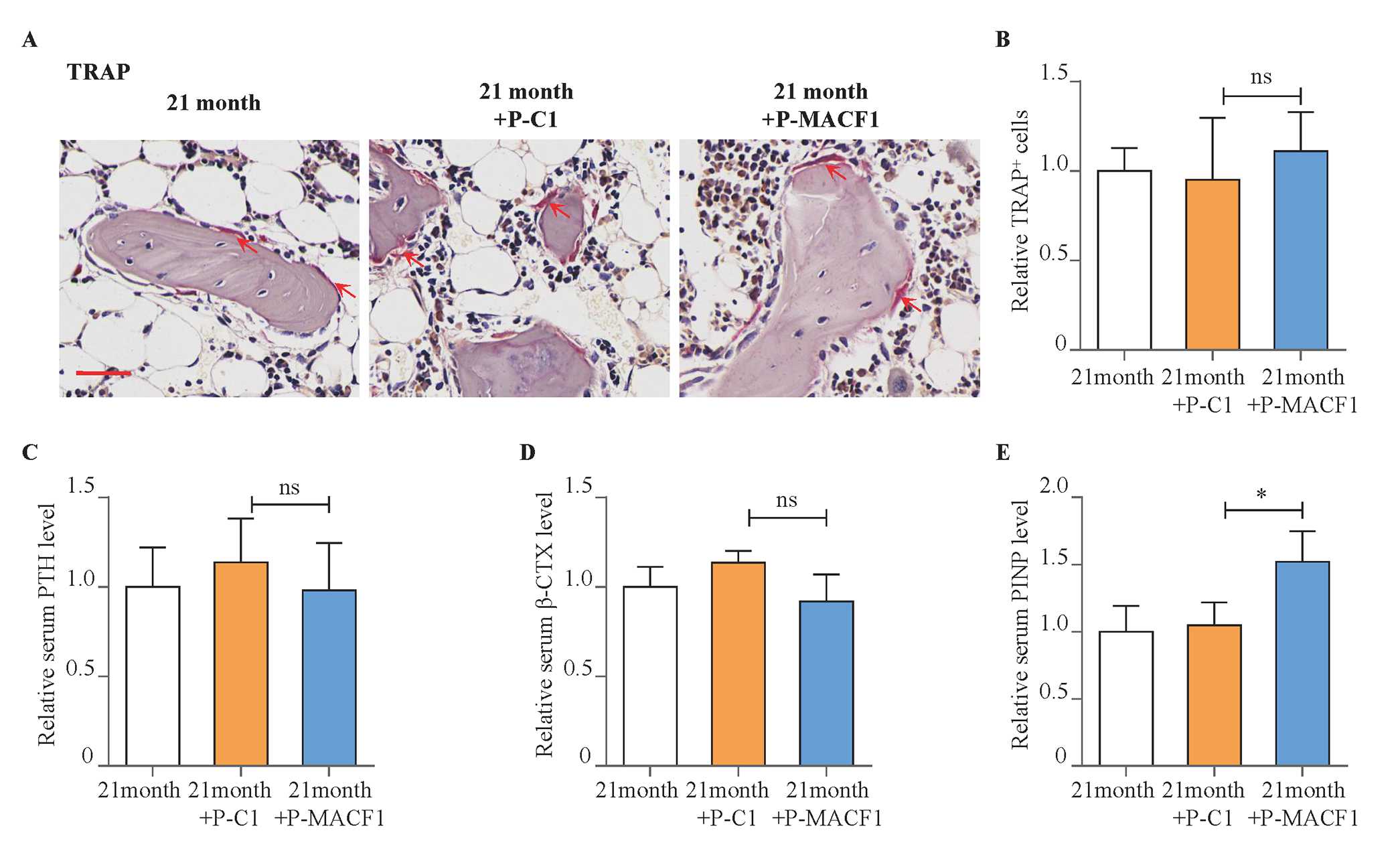


**Supplemental Figure 6. Effect of MACF1 on aging mice bone resorption**

**A.** TRAP activity in femur tissues of aging C57BL/6 mice distal femur after MACF1 over-expression plasmid treatment, as detected by TRAP staining. Scale bar: 50μm. 21month: 21-month control group. 21month+P-C1: blank plasmid treated group. 21month+P-MACF1: MACF1 over-expression plasmid treated group.

**B.** Relative number of TRAP^+^ cells, counted using Image-Pro Plus 6.0 software (mean ± SD).

**C-E.** Relative serum parathormone (PTH), β-C-telopeptides of type 1 collagen (β-CTX) and type 1 amino-terminal propeptide (PINP) of aging C57BL/6 mice after MACF1 over-expression plasmid treatment, as detected by electrochemiluminescence (mean ± SD, **P*<0.05).


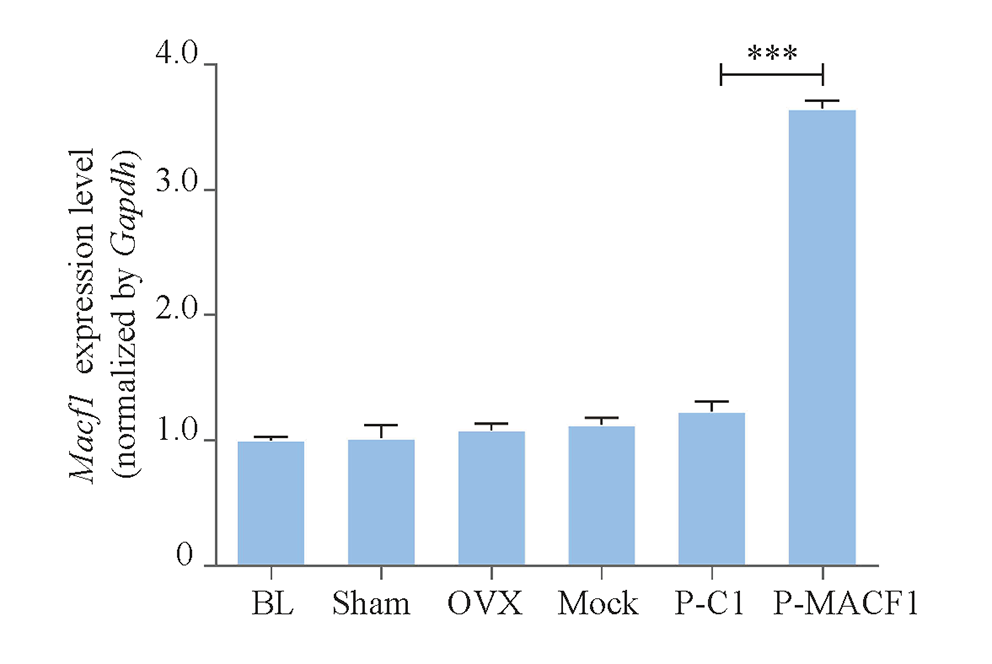


**Supplemental Figure 7.** *Macf1* expression levels of C57BL/6 mice after OVX treatment and MACF1 over-expression plasmid transfection, as detected by RT-PCR (mean ± SD, ****P*<0.001). BL (Baseline): sacrifice before siRNA treatment. Sham: sham OVX operation group. OVX: OVX group. Mock: transfection reagent control group. P-C1: blank plasmid treated group. P-MACF1: MACF1 over-expression plasmid treated group.

**
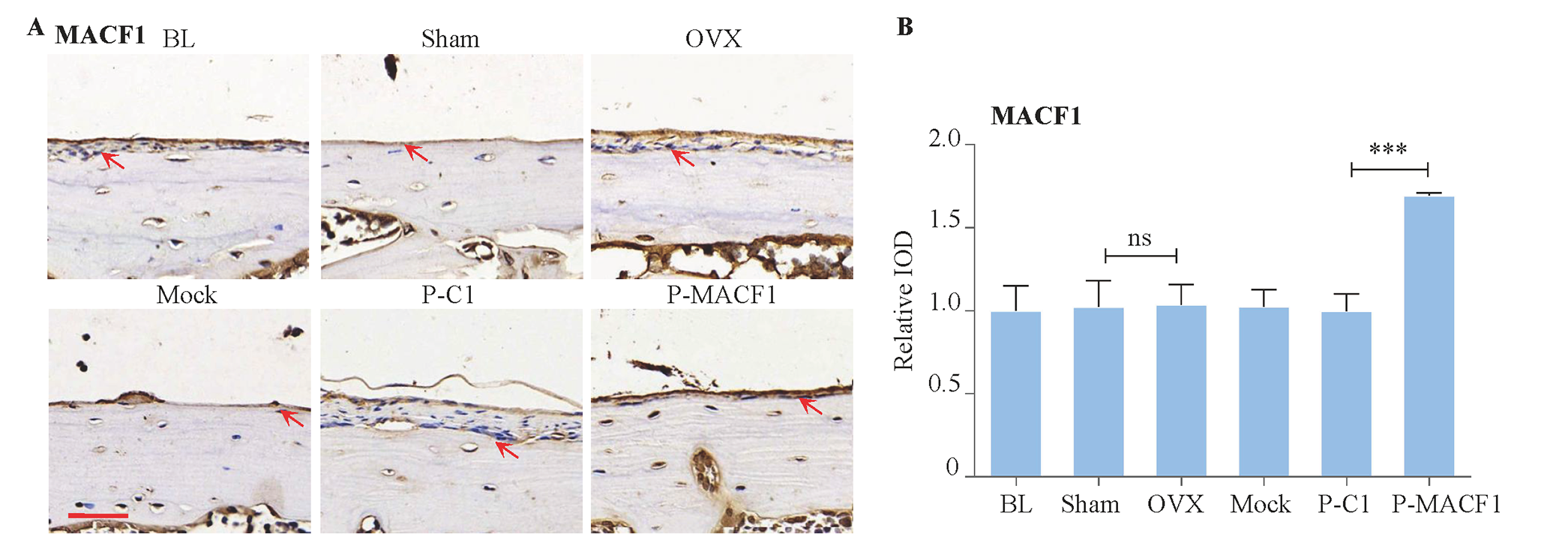
Supplemental Figure 8. MACF1 expression levels of C57BL/6 mice after OVX treatment and MACF1 over-expression plasmid transfection**

**A.** Expression of MACF1 in calvarial tissues of C57BL/6 mice after OVX treatment and MACF1 over-expression plasmid transfection, as detected by immunohistochemical staining. BL (Baseline): sacrifice before MACF1 over-expression plasmid treatment. Sham: sham OVX operation group. OVX: OVX group. Mock: transfection reagent control group. P-C1: blank plasmid treated group. P-MACF1: MACF1 over-expression plasmid treated group. Scale bar: 50μm.

**B.** Quantification of relative integrated optical density (IOD) values of MACF1 immunostaining using Image-Pro Plus 6.0 software (mean ± SD, ****P*<0.001).
